# The effects of corn silk on glycaemic metabolism

**DOI:** 10.1186/1743-7075-6-47

**Published:** 2009-11-23

**Authors:** Jianyou Guo, Tongjun Liu, Linna Han, Yongmei Liu

**Affiliations:** 1 Key Laboratory of Mental Health, Institute of Psychology, Chinese Academy of Sciences, Beijing 100101, PR China; 2 College of Food and Bioengineering, Shandong Institute of Light Industry, Jinan 250353, PR China; 3 School of Pharmacy, Shandong University of Traditional Chinese Medicine, Jinan 250355, PR China; 4 Molecular Biology Laboratory of Guang'anmen Hospital, China Academy of Chinese Medical Sciences, Beijing 100053, PR China

## Abstract

**Background:**

Corn silk contains proteins, vitamins, carbohydrates, Ca, K, Mg and Na salts, fixed and volatile oils, steroids such as sitosterol and stigmasterol, alkaloids, saponins, tannins, and flavonoids. Base on folk remedies, corn silk has been used as an oral antidiabetic agent in China for decades. However, the hypoglycemic activity of it has not yet been understood in terms of modern pharmacological concepts. The purpose of this study is to investigate the effects of corn silk on glycaemic metabolism.

**Methods:**

Alloxan and adrenalin induced hyperglycemic mice were used in the study. The effects of corn silk on blood glucose, glycohemoglobin (HbA1c), insulin secretion, damaged pancreatic β-cells, hepatic glycogen and gluconeogenesis in hyperglycemic mice were studied respectively.

**Results:**

After the mice were orally administered with corn silk extract, the blood glucose and the HbA1c were significantly decreased in alloxan-induced hyperglycemic mice (p < 0.05, p < 0.01, respectively), while the level of insulin secretionn was markedly elevated in alloxa-induced hyperglycemic mice (p < 0.05). The alloxan-damaged pancreatic β-cells of the mice were partly recovered gradually after the mice were administered with corn silk extract 15 days later. Also, the body weight of the alloxan-induced hyperglycemic mice was increased gradually. However, ascension of blood glucose induced by adrenalin and gluconeogenesis induced by L-alanine were not inhibited by corn silk extract treatment (p > 0.05). Although corn silk extract increased the level of hepatic glycogen in the alloxan-induced hyperglycemic mice, there was no significant difference between them and that of the control group(p > 0.05).

**Conclusion:**

Corn silk extract markedly reduced hyperglycemia in alloxan-induced diabetic mice. The action of corn silk extract on glycaemic metabolism is not via increasing glycogen and inhibiting gluconeogenesis but through increasing insulin level as well as recovering the injured β-cells. The results suggest that corn silk extract may be used as a hypoglycemic food or medicine for hyperglycemic people in terms of this modern pharmacological study.

## Background

Corn silk (ZeamaysL.) refers to the stigmas from the female flowers of maize. Fresh corn silk resembles soft silk threads 10-20 cm long that are either light green or yellow-brown in color. Corn silk contains proteins, vitamins, carbohydrates, Ca, K, Mg and Na salts, fixed and volatile oils, steroids such as sitosterol and stigmasterol, alkaloids, saponins, tannins, and flavonoids [1-14]. There have been many reports on the biological activities of corn silk constituents. Methanol extracts of corn silk showed an antioxidative activity on the level of lipid peroxidation [15]. Volatiles from corn silk inhibited the growth of Aspergillus flavus, indicating that it has an antifungal activity [16]. In addition, extract of corn silk inhibited TNF and LPS-induced cell adhesion, but not cytotoxic activity or TNF production [17].

Corn silk has been used in many parts of the world for the treatment of edema as well as for cystitis, gout, kidney stones nephritis and prostatitis [13,18]. Base on folk remedies, corn silk has been used as an oral antidiabetic agent in China for decades. However, in spite of its widespread use, the mechanisms underlying hypoglycemic activity of corn silk was not yet understood. Therefore, the purpose of this study was to investigate the effects of corn silk on glycaemic metabolism. The effects of corn silk on blood glucose, HbA1c, insulin secretion, damaged pancreatic β-cells, hepatic glycogen and gluconeogenesis in hyperglycemic mice were studied respectively.

## Materials and methods

### Animals

Kunming strain mice weighing 20-22 g, Grade II, Certificate SCXK (Lu) 20080006, were purchased from the Experimental Animal Center, Shandong University, China. The mice were maintained at room temperature under alternating natural light/dark photoperiod, and had access to standard laboratory food and fresh water *ad libitum*. This study was performed in accordance with the Guide for the Care and Use of Laboratory Animals. Care was taken to minimize discomfort, distress, and pain to the animals.

### Chemicals

Alloxan and adrenaline were analytical grade. Alloxan was purchased from Sigma Co., Ltd and adrenaline was purchased from Tianjin Amino acid Co., Ltd. China. Xiaoke pills were purchased from Jilin Liuhe Pharmaceutic Factory, China. Xiaoke pill is a kind of Chinese medicine used in the treatment of diabetes. It is composed of glibenclamide and several traditional Chinese herbs, including Radix Puerariae, Radix Rehmannia, Radix Astragali, Radix Trichosanthis, Corn Stigma, Fructus Schisandrae and Rhizoma Dioscoreae.

### Corn silk extract (CSE) preparation

Corn silk was obtained from local market. Sample was produced by the way introduced by Velazquez [1]. Briefly, corn silk was dried at room temperature (24.2 ± 1.0°C) and an aqueous extraction was performed by adding 100 ml boiling water to 10 g corn silk, filtering after 20 min and then lyophilizing.

### Experimental design

#### Blood samples from alloxan-induced hyperglycemic mice

One hundred mice were fasted for 12 h and then injected (iv) with alloxan (75 mg/kg) dissolved in sterile saline [19]. Forty-eight hours later, blood samples were collected from the tail veins of the mice. The blood glucose was analyzed with a Glucometer-4 (Bayer). Sixty hyperglycemic mice (the blood glucose level greater than 11.1 mmol/L) were selected and randomly divided into 6 groups. From then on, the 6 groups of mice were administered orally saline (control), Xiaoke Pill and 0.5, 1.0, 2.0 or 4.0 g/kg body wt. of corn silk extract (CSE) dissolved in the same amount of saline (experimental). The body weights of the mice were measured on the 0^th ^day, 5^th ^day, 10^th ^day, 15^th ^day and the 20^th ^day. At the same time, after fasting the mice for 12 h on the 20^th ^day, blood samples were obtained from the tail veins to determine the blood glucose levels. On the 45^th ^day, blood samples were collected from the orbital veins to measure the HbA1c with the HbA1c Apparatus (Variantα, Bio-Rad Laboratories) and insulin with an enzyme-linked immunosorbant assay (ELISA) kit (Biosource, Europe) respectively [20]. Then, the mice were sacrificed. The pancreas was dissected out and placed in 10% buffered formalin and the liver was dissected out for the measurement of hepatic glycogen. Estimation of the damaged pancreatic β-cells

#### The pancreatic tissues were embedded in paraffin blocks after formalin fixation

Paraffin sections were cut at 4- μm thickness and were deparaffinized in xylene twice for 5 min and then were rehydrated with the graded ethanol. The sections were examined after hematoxylin and eosin (H&E) staining [21].

#### Estimation of hepatic glycogen

The liver was homogenized in ice-cold 0.6 M HClO_4_. The mixture was immediately centrifuged at 3000 g for 10 min at 4°C to obtain the supernatant. Free glucose in the tissue was measured with the glucose oxidase method. Amyloglucosidase solution (10 U/ml) in 0.2 M sodium acetate buffer (pH 4.8) was then mixed and incubated in the mixture at 40°C for 2 h. After incubation, pH of the mixture was adjusted to 7 and subjected to determination of total glucose. Free glucose was subtracted from total glucose to obtain glycogen content. The glycogen was expressed as mg/g wet tissue [22].

#### Estimation of gluconeogenesis

Thirty normal mice were selected and allocated equally into 3 groups: Xiaoke Pill-treated group, CSE-treated group and saline group used as the control group. From then on, these 3 groups of mice were administered orally with Xiaoke Pill, CSE (4.0 g/kg) and saline respectively. At the end of the experimental period (15 days later), animals were fasted 12 h. After administration 1 h later, the mice were injected (s.c.) with L-alanine. The blood samples from the tail vein of the mice were collected at the 0^th ^min and 60^th ^min to determine blood glucose level.

#### Blood samples from adrenaline -induced hyperglycemic mice

Sixty healthy mice were allocated equally into 6 groups. Then these 6 groups of mice were also orally administered with saline, Xiaoke Pill and CSE (0.5, 1.0, 20. and 4.0 g/kg), respectively. On the 14^th ^day, they were fasted over night. After administration 1 h later, animals were injected (sc) with adrenaline. Blood samples from the tail vein of the mice were collected at the 0^th ^min and 60^th ^min to determine blood glucose level just as above.

### Statistical analysis

All data were analyzed by a one-way analysis of variance, and the differences between means were established by Duncan's multiple-range test [23]. The data represents means and standard deviations. The significant level of 5% (p <*0.05) *was used as the minimum acceptable probability for the difference between the means.

## Results and Discussion

Alloxan and streptozotocin are the most prominent diabetogenic chemicals in diabetes research. Both are toxic glucose analogues that preferentially accumulate in pancreatic beta cells via the GLUT2 glucose transporter [24-28]. Streptozotocin is split into its glucose and methylnitro-sourea moiety. Owing to its alkylating properties, the latter modifies biological macromolecules, fragments DNA and destroys the beta cells, causing a state of insulin-dependent diabetes (type 1 diabetes mellitus) [27,28]. On the other hand, alloxan has two distinct pathological effects: it selectively inhibits glucose-induced insulin secretion through specific inhibition of glucokinase, the glucose sensor of the beta cell, and it causes a state of insulin-dependent diabetes by selective necrosis of beta cells in type 1 and type 2 diabetes mellitus [24-26]. So alloxan is the agent of choice for induction of diabetic experimental animals in this study.

**Figure 1:**
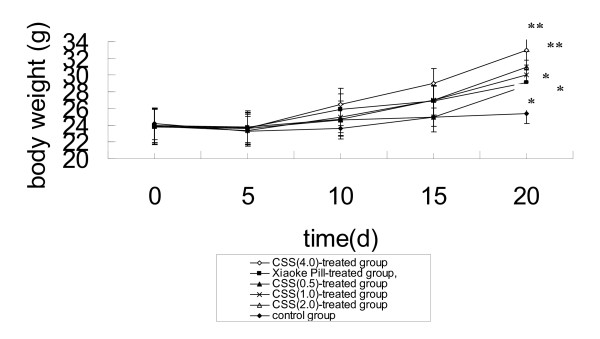
**Effects of CSE on body weights of hyperglycemic mice**. Values are means ± SEM, n = 10. *Different from control group P < 0.05; **different from control group, P < 0.01.

The body weights of the hyperglycemic mice induced by alloxan are presented in Fig. 1. Contrasted with the control group, the body weights of mice in CSE-treated group were increased gradually 20 days later (p < 0.05, p < 0.01). The body weights of mice in Xiaoke Pill-treated group were also increased. The body weights of hyperglycemic mice induced by alloxan were increased when they were administrated with CSE. It indicates that corn silk could act as supplement nutrients to mice.

**Figure 2:**
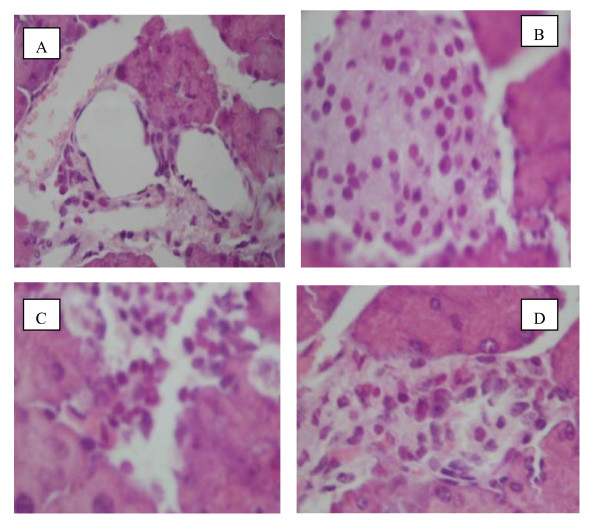
**Islet cell death and replication represented by hematoxylin--eosin**. The islet cells of diabetic mice of alloxan-treatment (**A**) showed extensive cell lysis, representing loss of plasma membrane with condensed nuclei and dissolved cytoplasm in wide intercellular spaces. In contrast, the islet cells of CSE(4.0)-fed mice (**B**) and Xiaoke Pill-treated (D) mice were partly recovered. The islet cells of CSE(2.0)-fed mice was C.

The results of blood glucose from hyperglycemic mice induced by alloxan are presented in Table 1. The levels of blood glucose were decreased after administration of CSE(2.0 and 4.0) and the Xiaoke Pill (p < 0.05). However, low doses CSE-treated groups (0.5 and 1.0 g/kg) did not reach the same result. The mechanisms of the hypoglycaemic effects of CSE have been also studied in this paper. As shown in Table 2, the levels of serum insulin were elevated after administration of 4.0 g/kg body wt CSE (9.8 ± 0.5 μU/mL p < 0.05) and the Xiaoke Pill (8.6 ± 0.7 μU/mL). However, the same results did not occur in the saline treated group(3.8 ± 0.4 μU/mL) throughout the total duration of the study. It is possible that CSE could aid in releasing of insulin from the surviving β-cells, as well from the recovered β-cells by CSE. The β-cells of the mice fed with CSE (4.0 g/kg) were partially recovered (Fig. 2). β-Cells death and alteration of islet cell population were prominent in the diabetic mice (Fig. 2A). In contrast, the cell damage was not observed in the islet cells of the CSE-fed mice in spite of the alloxan treatment (Fig. 2B).

**Table 1:** Effect of CSE on blood glucose levels in alloxan-hyperglycemic mice

Different groups	Blood glucose (mmol/L)
Control group	21.2 ± 2.1^b^
Xiaoke Pill-treated	13.4 ± 3.0^a^
CSE(4.0)-treated	11.5 ± 2.1^a^
CSE(2.0)-treated	15.6 ± 3.0^A^
CSE(1.0)-treated	18.9 ± 2.8^b^
CSE(0.5)-treated	19.9 ± 2.7^b^

Values are shown as means ± SEM, n = 10. The different letters in the same column indicate a statistical difference (*p *< 0.05)

**Table 2:** Effect of CSE on serum insulin level in alloxan-induced diabetic mice

Different groups	Serum insulin (μU/mL)
Saline-treated	3.8 ± 1.4^a^
Xiaoke Pill-treated	8.6 ± 0.7^b^
CSE (4.0)-treated	9.8 ± 0.5^b^

Values are shown as means ± SEM, n = 10. The different letters in the same column indicate a statistical difference. CSE-treated groups were compared with diabetic group, *p *< 0.05.

HbA1c is a more useful parameter in diabetes. CSE(4.0 g/kg) could decrease the concentration of HbA1c in plasma of alloxan- induced hyperglycemic group 45 days later (p < 0.01), as shown in Table 3. However the same result did not occur in other groups.

**Table 3:** Effect of CSE on HbA1c from hyperglycemic mice induced by alloxan (%)

Different groups	Results of HbA1c
Control group	11.8 ± 0.23^b^
Xiaoke Pill-treated	9.9 ± 0.30^b^
CSE(4.0)-treated	6.9 ± 0.28^a^
CSE(2.0)-treated	9.0 ± 0.26^b^
CSE(1.0)-treated	10.8 ± 0.33^b^
CSE(0.5)-treated	11.0 ± 0.22^b^

Values are shown as means ± SEM, n = 10. The different letters in the same column indicate a statistical difference (*p *< 0.01)

Adrenaline activates glycogenolysis and glyconeogenesis to elevate serum glucose level [29] and its effect is relatively rapid [30]. The results of blood glucose from hyperglycemic mice induced by adrenaline are presented in Table 4. It showed that ascension of blood glucose induced by adrenaline was not inhibited (p > 0.05) after mice were administered orally with CSE for 15 days. It indicates that the mechanisms of the hypoglycaemic effects of CSE might via increasing insulin level as well as recovering the injured β-cells but not through inhibiting gluconeogenesis and glycogenolysis. It is consistent with the results presented in Table 1, 3, 5 and 6.

**Table 4:** Effect of CSE on blood glucose levels in adrenaline -hyperglycemic mice

Different groups	Blood glucose (mmol/L) at 0^th ^min	Blood glucose (mmol/L) at 60^th ^min
Control group	5.7 ± 2.6	15.1 ± 1.0
Xiaoke Pill-treated	5.6 ± 2.2	12.3 ± 0.6*
CSE(4.0)-treated	5.8 ± 3.1	12.6 ± 1.5*
CSE(2.0)-treated	5.6 ± 2.6	14.0 ± 1.2*
CSE(1.0)-treated	5.7 ± 2.3	13.9 ± 1.6*
CSE(0.5) -treated	5.7 ± 3.3	15.0 ± 3.2*

Values are shown as means ± SEM, n = 10. * *p *> 0.05 vs. Control group

**Table 5:** Effect of CSE on hepatic glycogen

Different groups	Hepatic glycogen (mg/g tissue)
Saline-treated	14.2 ± 3.4
Xiaoke Pill-treated	16.8 ± 0.5*
CSE (4.0)-treated	17.0 ± 4.2*

Values are shown as means ± SEM, n = 10. * *p *> 0.05 vs. Saline - treated group.

**Table 6:** Effect of CSE on Gluconeogenesis

Different groups	Blood glucose (mg/mL) at 0^th ^min	Blood glucose (m/mL) at 60^th ^min
Xiaoke Pill-treated	92.5 ± 15.5	99.8 ± 11.9*
CSE (4.0)-treated	92.1 ± 17.9	102.6 ± 9.0*
Saline-treated	91.6 ± 14.7	100.0 ± 12.2

Values are shown as means ± SEM, n = 10. * *p *> 0.05 vs. Saline - treated group.

Glycogen storage in the liver is another way to maintain blood glucose concentration in mammals. Decreased hepatic glucose production is induced by glycogen synthesis and the treatment effects of CSE were further confirmed by the assay of evaluating glycogen storage in the liver. CSE treatment increased the level of hepatic glycogen. The glycogen levels were 17.0 ± 4.2 mg/g tissue in CSE treated mice. Concentrations of hepatic glycogen were lower in saline-treated mice (14.2 ± 3.4 mg/g) than those in CSE treated mice(Table 5). However, there was no significant difference between them (p > 0.05).

As shown in Table 6, At the 60^th ^min, the level of blood glucose of the mice in the CSE group was increased from 92.1 ± 17.9 mg/mL to 102.6 ± 9.0 mg/mL at the 60^th ^min, after the mice were injected (s.c.) with L-alanine. At the same time, the level of blood glucose of the mice in the control group was increased after the mice were injected (s.c.) with L-alanine (from 91.6 ± 14.7 mg/mL to 100.0 ± 12.2 mg/mL). The result was shown in Table. 6 (p > 0.05). Gluconeogenesis is one of the key metabolic pathways in the liver. It is an important mechanism for maintaining blood glucose within a normal range. The result shown in Table. 6 indicates that the mechanisms of the hypoglycaemic effects of CSE were not by inhibiting gluconeogenesis.

## Conclusion

Our results showed that CSE treatment markedly reduced hyperglycemia in alloxan -induced diabetic mices. The action of CSE on glycaemic metabolism is not via increasing glycogen and inhibiting gluconeogenesis but through increasing insulin level as well as recovering the injured β-cells.

## Competing interests

The authors declare that they have no competing interests.

## Authors' contributions

All authors were involved in the design of this study; and performed laboratory analyses and statistics. JG drafted the manuscript along with the other authors. All authors read and approved the final manuscript.
